# Draft Genome Sequences of Three *Capnocytophaga canimorsus* Strains Isolated from Healthy Canine Oral Cavities

**DOI:** 10.1128/genomeA.00199-15

**Published:** 2015-05-28

**Authors:** Pablo Manfredi, Francesco Renzi, Guy R. Cornelis

**Affiliations:** a Biozentrum der Universität Basel, Basel, Switzerland; b University of Namur, Namur, Belgium

## Abstract

Here, we present the draft genome sequences of three strains of *Capnocytophaga canimorsus*, each isolated from a different dog's mouth. Genome analysis provided evidence that these organisms may belong to a different nonpathogenic subtype of *C. canimorsus*.

## GENOME ANNOUNCEMENT

*Capnocytophaga canimorsus* is a capnophilic gliding Gram-negative bacterium from the *Bacteroidetes* phylum that is part of the normal flora of a dog’s mouth (1, 2). It is not reported to cause infections in dogs, but it causes severe infections in humans who are in contact with dogs (3, 4). It has been estimated by culture-based methods that more than every other dog carries *C. canimorsus* in its normal oral flora (5). Because of the very specific culture conditions required by *C. canimorsus* strains, their prevalence has often been underestimated. A recent study using PCR-based methods reported that up to 74% of dogs carry *C. canimorsus* in their mouths (6). Interestingly, despite such a high prevalence, human infections remain very rare. Considering that in barely half of the reported cases, patients do not show any obvious risk factors (7), it is possible that different strains are not equally virulent and that only a tiny proportion is actually pathogenic for humans.

The three strains CcD38, CcD93, and CcD95 were isolated from canine oral swabs and partially characterized (5). The strains were selected as potential representatives of a subgroup of *C. canimorsus*, as suggested by 16S rRNA phylogenetics and various phenotyping assays (5, 8). Genomic DNA was extracted using the Genomic-tip 500/G DNA extraction kit (catalog no. 10262; Qiagen), according to the manufacturer’s instructions, followed by an additional phenol-chloroform purification step. Sequencing was performed at LGC Genomics, Berlin, Germany, on one Illumina HiSeq 2000 channel and generated between 8.6 and 9.8 million 100-bp single reads per strain. Due to low mapping efficiency on the *C. canimorsus* 5 reference genome (9), *de novo* assembly was performed with Velvet with optimized *k*-mers (10). The sizes of the draft assemblies ranged from 2.56 to 2.74 Mb, with 191 (CcD95), 216 (CcD38), and 293 (CcD93) contigs. Genome annotation and preliminary analyses were performed by the LABGeM, France Génomique (11). The global G+C content (35.56% ± 0.07%) differs slightly but significantly from that of clinical isolates of *C. canimorsus* (36.16% ± 0.08%; *P* < 0.0002). A total of 2,325 to 2,618 coding sequences (CDSs) were identified per genome, giving a core genome of 1,896 orthologous groups. When considering the whole *Capnocytophaga* pangenome, 290 genes were conserved and exclusively found in the three genomes presented here. This is similar to the number of *Capnocytophaga cynodegmi*-specific genes (341) (12) and supports the idea of a speciation event within the *C. canimorsus* taxon. Besides 247 clusters of unknown function, 16 genes implicated in basal energy metabolism, 10 in polysaccharide synthesis, 11 in integral outer membrane proteins, and 5 in ABC transporters formed predominant functional classes of the species-specific core. In addition, a consensual phylogenetic tree based on 771 individual phylogenies of orthologous proteins conserved among all *Capnocytophaga* spp. exhibited a very clear discrimination between clinical (clustering together in 677 trees) and canine oral (clustering together in 729 trees) isolates of *C. canimorsus*, even compared to a different species, such as *C. cynodegmi* (clustering together in 686 trees). This is again consistent with a potential speciation event within the *C. canimorsus* taxon.

### Nucleotide sequence accession numbers. 

These whole-genome shotgun projects have been deposited in ENA under the accession numbers CDOI00000000 (CcD38), CDOL00000000 (CcD93), and CDOH00000000 (CcD95). The versions described in this paper are the initial versions.
